# Military Surgery

**Published:** 1845-01

**Authors:** 


OUTLINES OF MILITARY SURGERY. By Sir *George Ballingall*, M.D. F.R.S.E. Third Edition. 1844.

RECUEIL DE MEMOIRES DE MEDICINE, DE CHIRURGIE, ET DE PHARMACIE MILITAIRES. Vol. 56, Paris, 1844.

A Collection of Memoirs on Military Surgery, Medicine and Pharmacy. Vol. 56.

A MEDICAL work reaching its third edition can require little notice or recommendation upon the part of the Reviewer; but we may observe that, we think its value would have been enhanced if the first division, relating to what may be termed Military Hygiene, had formed a larger proportionate share of its contents. This, however, could only be accomplished by much enlarging the size of the book, or by trenching upon the purely surgical portion, which the author considers as specially demanding his attention.

"So long as this course stands alone, the only one of its kind delivered in the medical schools of the united kingdom, so long it can only be patronized and encouraged as a substitute for part of the surgical attendance enjoined by the several colleges: and hence I feel myself bound, in justice to my pupils, to enlarge upon the surgical department. Again, I find that this is the department of the course which proves most interesting to that important part of my audience consisting of gentlemen returning from foreign service, to refresh their memories, or to renovate their knowledge at the schools of medicine. * * * * * * * * * Without at all underrating the importance of those diseases more strictly within the province of the physician, I cannot avoid expressing my fears, that in a period of long protracted peace, there is some risk of the medical officers of the army overlooking the importance of the surgical department of their profession."

That the army or naval medical officer should be made fully acquainted with every surgical duty which he, beyond any of the profession, is most likely to be called upon to fulfil, is too obvious to require farther remark; but it is no less true that this forms but a portion, and even a limited portion, of what should be expected from him, and is by far the most easy of attainment at the ordinary courses of surgical instruction at our schools. Such knowledge will, indeed, prove invaluable upon the field of battle: and it is a matter of sincere congratulation that the gross ignorance which disgraced the lower ranks of medical officers of both the French and British armies at the commencement of the late wars can never be again witnessed. But do not the histories of all campaigns and naval expeditions, and the defective health and large mortality of our soldiers and seamen even during a period of profound peace, too truly indicate that sources of destruction far more potent and more constant in their operation than the battle-field are in existence? Formidable as these evils are, they are still very much diminished, for even the condition of the warrior has participated in the general progress of improvement. But such improvement has only arisen from the diffusion of more sound principles of hygienic treatment, and can only be augmented by their still farther dissemination. Where is the young medical officer to seek for the necessary information upon this subject if not at the hands of his experienced teachers of military medicine; for it is obvious that, at the ordinary courses of the schools, none such can be supplied. It is true that we possess a great number of works which incidentally convey most valuable information upon this head, and much of modern improvement is due to their publication: but we have no work treating Military Hygiene as a substantive subject, although, from its great national importance, it is worthy of the employment of the highest talent and most extensive experience. We sincerely hope the Head of the Army Medical Department has not abandoned the intention the author states he once entertained of producing such a one.

In truth there is no member of society, placed above destitution, whose condition is more worthy of consideration and commiseration than that of the common soldier. Frequently entrapped into the service of the public at an unwary moment, he discovers, when too late, that he has contracted an interminable bondage with a hard task-master. Neither emancipation nor advancement stimulates and rewards good conduct, while the smallest delinquencies are visited by punishments determined by an arbitrary system of laws, and too often inflicted with most injudicious severity. It might be expected that the complete abrogation of the moral judgment, and the conversion of the man into an intelligent machine, which military discipline exacts, would, at least, be compensated for by a due provision for every physical comfort, as is seen to be the case in some of the other more avowed forms of slavery. With the soldier it is not so: for, whether we regard his clothing, his diet, his abodes, his recreations, his nightly rest, the due alternations of moderate employments with proper intervals of repose, or a variety of other circumstances, we see cause to acknowledge the necessity of great ameliorations before his hygienic condition can be considered satisfactory. Many of the inconveniencies of a military life are indeed inseparable from it during the period of war, although even then susceptible of great mitigation: but the extent to which they are allowed to continue during peace is lamentable. What are the results of the conjoined operation of the various circumstances unfavorably influencing the condition of the soldier? A large proportion of premature deaths and suicides, frequent attempts at desertion, an addiction beyond all other classes to intoxicating liquors, and the generation of such a host of fictitious maladies, that no small share of the duties of the military surgeon, aided by a now distinct branch of medical literature, consists in their detection.

We proceed to notice a few of the observations made by Sir George Ballingall upon this subject.

*Diet.*—The importance of a wholesome and abundant diet, as a means of preservation of the health of the soldier, is now almost universally acknowledged. Dr. Jackson, however, believes that a frugal fare has usually been that of the conqueror, and a rich and luxurious one that of the conquered.

" In his last work on the 'Formation, Discipline, and Economy of Armies,' Dr. Jackson has adduced much ingenious argument in support of his views of this subject; and he concludes by observing, that luxurious living places the military character on the brink of destruction; 'for,' says he, 'if there be anything like correct observation among men, it may be confidently asserted that, if high living be the life of the gentleman, it is the death of the soldier.' It is obvious, however, that such observations as the foregoing apply only to the luxuries occasionally indulged in by the higher ranks. They can only be addressed with propriety to the officers—to the educated and reflecting part of the army—and habits of abstinence are so little congenial to the disposition of the English soldier, that he will never practise them when he can do otherwise. My views of the disadvantages of an insufficient diet are, I find, fully borne out by the recent experience of Mr. Alcock of the British Legion, who, in a letter to me, observes that his men, at Vittoria, were literally starved upon their rations, nominally a pound and a half of bread, and a pound of meat; but this, when served to the soldier, after the peculations, and diminutions from bone and skin, was often not more than four or six ounces of solid meat. 'This,' says he, 'is not sufficient, and formed a very prominent cause of a startling mortality.' " 37.

Where practicable, daily rations are to be preferred as preventing the waste and gluttony which sometimes are present when several days' are delivered out at once. In a letter to Sir James M'Grigor, the Duke of Wellington states that rations were invariably delivered daily in the Peninsula; and that the British soldier carried but three days' bread, while the Portuguese carried six, and the French fifteen days'. Almost all observers agree in the importance they attach to the introduction of tea or coffee for the soldiers' breakfast. Their use seems often prophylactic in cases of exposure to malaria, night air, &c. The benefits resulting from substituting these drinks for spirits in both Army and Navy are acknowledged on all hands. " Coffee," says M. Finot, in an article to which we shall presently advert, " now received in the berry and ground as wanted, forms the best of drinks for the French soldier in Africa. The advantages which have attended its replacing the former distributions of brandy, are, we believe, now definitively agreed upon."

*Clothing.*—We do not think the author has sufficiently exposed the defective character of this. To us it seems that it would be difficult to contrive more unsuitable habiliments than those of the soldier, which restrain by their tightness free muscular action, rendering the occasional active exercises painful and dangerous, and by their flimsy texture afford an insufficient protection against the inclemencies of the weather. White trowsers may look very nice upon parade, and please the eye of the commanding officer, but, in this and other cold and inclement climates, should never be employed, especially when it is considered that it is impossible for the soldier to keep them sufficiently clean without the use of wet pipe-clay. Under-clothing of every description, both waistcoats and drawers, is also far too much neglected, although instances of the prevention and removal of disease by its adoption are exceedingly numerous. When we consider the vicissitudes of weather a soldier is exposed to, his alternations of active exercise and perfect inactivity, and the broken description of rest he obtains, it seems evident that he is very insufficiently clad, and that above all things he should be furnished with a better supply of flannel.

*Exercises.*—The author thinks that these require great attention to be paid to them by the officers, agreeing with Sir John Pringle, that "although a soldier is occasionally liable to great fatigue, the most frequent error of people of that class, if left to themselves, would be on the side of rest." The most eligible rate for marching in all climates is from three to three and a half miles per hour.

"I have been much pleased to find my own views on this point strongly confirmed by a recent authority, Dr. Kennedy, of the Bombay army, who, in his interesting 'Campaign of the Army of the Indus,' observes, that 'when the soldier has from 50 to 60 pounds weight to carry, a distance of 12 or 14 miles to march, and a solar temperature above 100° to bake in, the shorter the time he is about it the better.' It is in my opinion an erroneous practice, as was the custom during my service in India, from fear of exposure to the sun, to move off the troops from the ground at two or three o'clock in the morning, sometimes even earlier. Their sleep was thus broken; and the march, accomplished in the dark, frequently over bad roads or no roads at all, was necessarily protracted, was attended with great additional fatigue, and not without risk to the limbs of individuals. An hour's more sleep, and an hour's more daylight, even at the expense of an hour's more sun, was the maxim which I endeavoured to inculcate." 46.

*Accommodation of Troops.*—We cannot compress the few remarks the author makes upon the construction of camps. He particularly insists that, when these prove unhealthy, other localities should at once be chosen, rather than attempts be made, which usually fail, at remedying the evil. In the construction and regulation of *barracks*, means for *thorough ventilation* and perfect *cleanliness* should be carefully provided for. "The class of society from which soldiers and their wives are taken have an incorrigible aversion to the free circulation of air, a circulation which is rendered more necessary for them than for the higher ranks, in consequence of their less minute attention to personal cleanliness." Dr. Reid's Illustrations of Ventilation, noticed in a recent number, supply us with numerous examples of the defective condition of shipping as regards ventilation. Sir George Ballingall describes an easy and effectual mode of ventilating barracks, by which the currents of air so detrimental to health and comfort are avoided. Dry-scrubbing the floors has been advantageously substituted in numerous instances for the former too frequent washings. This cannot always be done.

"In warm climates, where vermin, and particularly fleas, are abundant, frequent washing is perhaps a necessary resource. Mr. Alcock tells me, that in the large hospital at San Telmo, containing 600 beds, he had each division scrubbed with water every morning at day-break during the summer-months, to the manifest comfort of the patients, who were thus kept free from vermin, greater coolness and cleanliness were obtained, and no bad result was observed. By the general introduction of iron bedsteads into barracks, cleanliness has been essentially promoted, and ventilation improved; for those cumbersome wooden bedsteads formerly in use, not only obstructed the circulation of air, but even materially diminished the cubical bulk of it contained in a room."

The importance of *whitewashing* hospitals and other crowded receptacles is now generally acknowledged, and the author deplores the unnecessary obstacles and delays which often prevent its execution, as well as the imperfect manner in which it is usually performed. We have often regretted the remissness of parochial authorities in applying this useful preventive of infection to our wretched alleys and courts; but the neglect in the case of barracks, military hospitals, &c., where all the appliances and plenty of hands may be easily furnished, is far more culpable.

*Proportion of Sick in Armies.*—This may be much diminished by careful attention to the various circumstances mentioned. As already observed, those who fall in battle constitute but a small portion of the total mortality. Sir D. Stewart states, "that the 92nd regiment lost more officers and men in four months from the climate of Jamaica, than by the hand of an enemy in an active war of 22 years, in the progress of which it was 26 times in battle." After alluding to the large proportion of sick reported by Pringle, in the campaigns of the Low Countries, the author observes—

"To compare this with what has recently happened in another quarter of the world, I may notice the statement made regarding the sickness in the Bombay division of the Army of the Indus. From 1st Nov. 1838 to 31st Dec. 1839, the number treated was, Europeans and Natives, 11,689, and the deaths 408, which, compared with the strength, sufficiently indicates the hardships endured, and the efficiency of the hospital establishments. From Col. Tulloch's statistical statements it is inferred that soldiers in the United Kingdom are under medical treatment once in 13 months; in the West Indies twice within that period. In this country 1 case in 67 proves fatal, and in the West Indies 1 in 24."

Mr. Edmonds states, from returns in the Adjutant-General's Office, that in the Peninsular Army, averaging 64,227, the annual ratio of mortality, in the years 1810-13, was 10 per cent. officers, 16 per cent. men, and that during this period there were constantly $22\frac{1}{2}$ per cent. sick. Vaidy, in his article "*Hygiene Militaire*" in the "*Dictionnaire des Sciences Medicales,*" states that, under the most favourable circumstances, an army will furnish about 5 per cent. of sick. During a campaign not less than 10 per cent. must be calculated, and in the event of reverses or other untoward circumstances, this becomes immensely increased. As an example of what may be effected by able medical superintendence, the author cites the following incident of the Peninsular war.

"During the ten months from the siege of Burgos to the battle of Vittoria, inclusive, the total number of sick and wounded which passed through the hospitals was 95,348. By the unremitting exertions of Sir J. M'Grigor and the medical staff under his orders, the army took the field, preparatory to the battle with a sick list under 5,000. For twenty successive days it marched towards the enemy, and in less than one month after it had defeated him, mustered within thirty men as strong as before the action, and this too without reinforcements from England, the ranks having been recruited by convalescents."

There are several interesting observations upon the construction and management of Military Hospitals, and the most approved modes of Transportation of the sick and wounded.

The other work noticed at the head of this article is the last volume of a series of papers upon Military Medicine, contributed by the medical officers of the French Army. Our neighbours, as is well known, have long been ardent cultivators in this field, and the labours of some of their most eminent observers are scattered over the half-hundred volumes of this Collection which have appeared. The principal paper in the present volume relates to

THE MEDICAL HISTORY OF ALGERIA, the acquisition of materials for the illustration of which, has long been an object of ardent desire of the Military Council of Health. This has been responded to with alacrity by the medical officers of the African army: and documents illustrating the topography of the various localities, and the nature of the different diseases which prevail in them, are continually arriving. The progress of the French soldier is represented as being constantly attended with an improved sanitary condition of the portions of the country subjected by his arms, and one important fact is deduced from the various reports.

"A fact beyond all doubt at present, is that the climate of Algeria is healthy, and that the disastrous epidemics which have thinned our armies are due to local causes susceptible of removal."

The present contribution is an account, by M. Finot, of the Military Hospital at Blidah, in 1842, during which he treated upwards of 8,000 patients there. It is a most interesting document, amply illustrated with tabular views of results, and we regret that our space will allow only of allusion to some few of the observations it contains.

The conquered Arabs in their ignorance reject the benefits which medicine would confer upon them.

"The apathy of Mussulmen during epidemics is well known. Nothing is more common than to see men and children, having small-pox pustules in full suppuration, lying in the mud in the streets of Blidah, and in the roads of its vicinity, scarcely covered by a few wretched rags. They refuse the benefits of vaccination, and it is only by deceiving them that we have been able to subject a few to its influence. Many of the numerous diseases arise from the frequent ablutions which their religion necessitates. To this cause, and to the habit of sleeping in the open air, must be attributed the very frequent occurrence of pulmonic affections among the Arabs, compared to the Europeans resident at Blidah."

When the more intelligent of the Arabs become affected with *small-pox*

they adopt a practice which they state diminishes the deformity, duration, and danger of the disease. As soon as the eruption commences, every part of the body is covered with pledgets previously macerated in oil. When the period of suppuration arrives, the patient is washed, cleaned, and again well greased.

Treating of Syphilis, M. Finot mentions the curious manners of the country as respects *prostitution.*

"This is not as with us a vice and object of detestation. It is a peculiar occupation, which a woman enters or leaves just as she feels disposed. Prostitutes are under the protection of the Mezouar, a kind of public officer somewhat resembling a French Commissioner of Police. He exercises over them an unlimited authority, which he often abuses for the extortion of money; but he will not allow any one else to offer the slightest violence to his *protegées.* When a woman suffers injury at the hands of her husband or her friends, she flies to the Mezouar, whence she can only be removed on the payment of a certain sum agreed upon among the parties. If this is not effected she becomes a prostitute, or, as it is called here, a 'free woman.' She now acquires a right to appear in the streets with her face uncovered, and takes a part in all festivities, without any one, even her own mother, venturing to interfere with her. When she desires to re-enter her former mode of life, or to marry, she presents herself before the *Cadi,* with two witnesses. There she declares she wishes to live in future like a good Mussulman. A copy of her declaration is supplied her, and no one possesses any right of inquiring as to her former mode of life. We know at least a dozen such girls who have thus quitted a life of debauchery, and who have married Moors well-to-do in the world. The Mussulmen do not look upon these prostitutes as degraded beings, since they allow them to go into the best society, and to frequent the baths with their own wives. They believe them foolish, or as actuated by an evil spirit."

Of 37 of these women who were affected with syphilis at the time of writing, the author states 32 were married, and 5 only single women. Thirty of the number declared they were driven to prostitution by poverty or ill-treatment.

*Two Medical Seasons.*—The number of admissions from July to December singularly preponderated over those from January to July. The periodical increase is very evident when stated in *trimestres* (by three months). Thus, in the *Plain of Mitidja,* the patients increased thus: 1st trimestre, 1401; 2nd, 2105; 3rd, 4314; 4th, 2058—July, August, and September being the endemo-epidemial months *par excellence.* The same constitution was observed in other portions of Algeria whose topographical and other conditions were very various. Taking the military hospitals of Algeria in 1841 generally, the numbers stand thus:—

| | | |
|---|---|---|
| 1st. Trimestre | 15,582 | =39,913 |
| 2nd. " | 24,331 | |
| 3rd. " | 50,937 | =83,379 |
| 4th. " | 32,442 | |
| | | 123,292 |

*Endemo-Epidemics.*—These are intermittent and remittent fevers of every type, periodical neuralgiæ, and diarrhœa and dysentery—affections however different in many respects, all springing from common causes, which often rendered their progress and treatment identical. Of every 100 patients admitted, 85 laboured under this class of affections, and but 15 of all other maladies. After they have passed away, the health of the soldiers often continues long in a debilitated state—a condition termed by the author *chloro-anæmia*: and of 745 chloro-anæmics above 100 became dropsical. While in the marshy districts of France the proportion of sporadic to intermittent diseases is about one-half, in Africa it is about one sixth.

*Relapses.*—The admissions into the hospitals of Algeria, 1841, amounted to 114,267, while the effective force was 75,000. "However high this figure may be, it does not indicate the proportionate number of diseases of our soldiers; for every soldier, prior to admission, had had quinine administered to him, sometimes five or six times. Upon enquiry of each patient on admission, we are astonished at the number of relapses, and it is remarkable that these are especially noticed after a sojourn in Africa for two or three years. In some individuals the relapses recur with such frequency, are produced by such slight causes, and prevented with so great difficulty, that they cannot properly be said ever to be free from the fever."

*Mortality.*—The mortality in the hospitals of Algeria, 1841, was 1 in 14.65, which is favorable, compared to that of the civil and military hospitals of France. The viability of the civilians is considerably less than in the most unhealthy localities of France. The deaths are 1 in 15, and of the indigenous tribes who neglect medical aid, 1 in 13. As to the influence of *age*, the endemo-epidemic affections are most potent from adolescence to 30, after which their power over the system decreases. The ages, 22, 23, 24, furnish the greatest amount of deaths: thus of 798 deaths, 545 occurred at or below 25, and 253 at or above 26. In estimating these numbers it must be remembered, however, that the former age is that of the great body of the recruits. "The army," says M. Finot, "in the majority of its individuals, offers exactly the conditions, as regards age, which are most favorable to the contraction of endemo-epidemic diseases."

*Diet and Regimen.*—The author dwells upon the importance of supplying good flour for bread, and the dealing out a sufficiency of meat, which he thinks the French soldiers do not obtain. He truly says, military rations are not usually sufficiently varied according to the different seasons of the year. Of *Regimen* he observes—

"The doctrines of Pringle upon the causes of the diseases of troops may be applied with perfect truthfulness to our troops in Africa. That which was true a century ago still continues so at the present time. Pringle maintained that fever, diarrhœa, dysentery, &c., result from atmospheric influences and seasonal vicissitudes, and are not for the most part the product of excess on the part of the soldier, nor of his use of bad water, or abuse of alcohol. Not but these causes may and do produce, in some individuals, great derangements of health, but these are individual cases only."

The abuse of spirits leads to diarrhœa and dysentery rather than fever. Excessive coitus, masturbation, *i. e.* whatever tends to prostrate the nervous energy, yields up the constitution defenceless to the causes of fever. Men who are well nourished, and especially such as have their intellectual powers regularly developed, usually resist influences to which others succumb. The author states confidently that, if recruits were not allowed to enter the African army until after the age of 25, at least one-third of the present mortality would be spared.

Upon the *Treatment* of the diseases of Algeria M. Finot observes :—

" 1. The expectant mode is that adopted by the natives. They patiently wait until it shall please God to rid them of the fever;—but, while waiting, their constitution becomes deteriorated, the process of nutrition perverted, and the viscera engorged; and they perish young and almost in a state of anæmia. 2. The purely antiphlogistic method is condemned by facts. 3. The evacuant method, employed exclusively, is inadmissible. 4. The exclusive administration of quinine may advantageously be substituted for any of these modes. 5. The mixed plan of treatment consists in the combined use of antiphlogistics, or evacuants and quinine. 6. Lastly, there is the eclectic mode of treatment, which, especially in the treatment of the endemo-epidemic affections, draws its indications from the season, the medical constitution of the locality, the age, sex, and constitution of the patient, the duration, type, and complications of the malady, the condition of the organs, &c.;—and having well considered and weighed all information so derived, employs as means of cure all the agents to which it has access, quinine, opium, as well as depletions, emetics, and revulsives."

A paper is contributed by Dr. Bruguière upon the medical topography of *Milianah*, for the purpose of exhibiting the sanitary effects of attention to all the requirements of hygiene, and the provision of good hospital accommodation. This town, captured by the French from the Arabs in 1840, was the one of all others in which the first garrisons suffered the greatest mortality, which was attributed to the influence of the sirocco, malaria, and similar causes. The effect of these from the locality of the place the author denies; and attributes the cruel ravages to the wretched accommodation offered to the troops, who were at the same time submitted to excessive labour under a burning sun, and could obtain but an insufficient supply of provisions. Under these circumstances the mortality, though dreadful, was not surprising. A garrison of 1200 furnished 2,217 admissions into such wretched hospitals as could be provided in the course of the first four months, and, in four months after, there were 676 deaths recorded. He presents a tabular view of admissions and deaths, showing, among other results, that while 2217 admissions in 1840 furnished, in four Summer months of 1840, 568 deaths, 572 admissions in the same months, and the improved condition of 1841, furnished but 44 deaths. The circumstances to which he attributes this improvement are the excellent supply of food, the cleansing the streets and roads, the construction of a good hospital, the moderate employment of the troops, and the provision for their recreations.

Respecting the *Sirocco*, Dr. Bruguière observes—

" The sirocco, or wind from the southern desert, which is said to have blown last year during forty consecutive days, and the influence of which we often feel, produces an ardent heat, which so instantly rarefies the air that breathing becomes panting and difficult. Although it exerts its influence upon all individuals, it does so in an especial manner upon those of a nervous or irritable temperament, in whom it produces a condition of general debility, accompanied by violent pains in the limbs. The organ it especially affects in these is the stomach, in which it induces a condition of atony, or indifference to the presence of food, which it either rejects or digests imperfectly, or a vehement craving for alcoholic and exciting drinks, which, however, when taken in moderation, calm its appetence and restore its energy. Prepared by the observations of my predecessor last Summer, I expected to find, during the early months of my residence in this town, that the Sirocco played a great part in the diseases which came under my observation, and I determined to study very attentively the nature of its influence upon their production, course, and termination. To the present time I have been able to make no other general observation than the one mentioned above, and I am almost tempted to believe that they have attributed to this wind a more evil influence than it really exerts."

The department of the Collection devoted to *Clinical Surgery* contains several interesting cases. We may advert to one or two of these. The first was a most extensive *gun-shot wound of the face* (suicidal), several of its bones being severely fractured; reparation, however, took place to a surprising extent, and that with very little constitutional disturbance. The patient was about to be discharged, ten weeks after his admission, when he complained of slight pains in the head, which quickly augmented in severity, and became accompanied by remarkable prostration of strength, and slowness of pulse (30). He died in four days, not having exhibited any lesion of intelligence, loss of sensibility, paralysis, or convulsion.

The examination proved, however, that mischief had long been going on within the brain, although untestified by any symptom, until the last three or four days. The entire right anterior lobe was in a state of pulpy *ramolissement*, and its interior surface converted into one vast abscess, extending beyond the fissura Sylvii, and communicating with the ventricles, into whose cavities, as well as into the vertebral canal, the pus had penetrated. Below, all cerebral matter was completely destroyed, the membranes alone separating the abscess from the bones. The internal surface of the left lobe, where it came in contact with the right, was also in a state of *ramolissement;* but no vascular injection or other alteration was observed in other parts of the brain. At the right side of the *crista galli* a perforation, communicating with the nasal fossa and frontal sinus, was discovered, large enough to admit the point of a finger. Looking into the nasal fossa through this aperture, a foreign body was observed to be lodged within it, which was easily removed by a forceps. It proved to be half a bullet, so that the ball must have split into two pieces, one of which, after effecting the perforation in the ethmoid, fell back again into the nasal fossa and there remained. No foreign body whatever was found within the cavity of the skull.

Another case was also an example of the insidious progress of a formidable disease. The limb of a patient, who was suffering from *necrosis of the ankle-joint,* was removed. The stump cicatrized, although slowly, but an obstinate diarrhœa, and an œdematous condition of the limb coming on, the patient eventually sank about two months after the operation. At the post-mortem an enormous abscess was discovered in the abdomen, in contact with the lumbar region of the spine. Five of the vertebræ, and a portion of the sacrum, were in a condition of extensive *caries*. The veins leading to the lower extremities were inflamed, and filled with coagula.

The medullary substance of the tibia of the amputated limb was also found in an inflamed condition, and upon the last point M. Marchal, commenting upon the case, thus observes :—

"We cannot too earnestly call attention to *osteo-myelitis* succeeding amputation, which is the unsuspected cause of death in a great number of fatal cases. Lately, the limb of a patient, under the care of M. Velpeau at La Charité, was removed for cancer of the knee. After some days he was seized with shivering, and then with the various symptoms characteristic of the ataxo-adynamic condition. The medulla projected like a mushroom from its canal, and many believed it to be a sudden reproduction of the cancer. Upon examination after death, the whole interior of the femur was found of a bright red from the point of its division."

The last case to which we shall allude is termed by its reporter, an example of *spina ventosa of the humerus* successfully treated by hydriodate of potash. The soldier was admitted into the hospital in 1841 with two-thirds of the superior portion of the left humerus in a state of great hypertrophy, and that especially where the middle and lower thirds of the bone are in contact. Pressure was attended with a slight crepitation. The tuberosities participated in the development. The skin was somewhat reddened and tense. The muscles free and unadherent, but diminished in size. Two years before, he had perceived a small tumour near the external tuberosity, the bone at the entire upper part of the arm shortly after enlarging. Suppuration formed in the little tumour, and a small quantity of matter was discharged from a fistulous opening from time to time, with little pain or irritation, the power of movement of the extremity being however considerably enfeebled. About a twelvemonth before his admission, the limb, which had remained very quiet, was struck against a table, after which the pain became much more severe, and the pus re-appeared and continued to be discharged.

The patient, who was of a lymphatic temperament, and scrofulous descent, was placed upon a tonic regimen, and hydriodate of potass was prescribed, mercurial frictions being applied to the arm. For some days after, the pain became much increased in severity, but not augmented by pressure. On the eighth day, the patient complaining of the excess of pain and more limited range of motion of the arm, M. Godard examining it, discovered that the humerus was fractured at the most prominent part of the hypertrophy, a little below the middle portion of the bone. The man was not aware of this accident, and only recollected that he suffered great pain upon awakening in the morning. The fracture was somewhat oblique, the superior fragment being drawn outwards and forwards, projecting prominently beneath the skin. The volume of the arm was increased, while its length which, prior to this complication, was slightly augmented, was as much diminished. The adjustment of the fracture caused excessive pain. Very firm pressure, by means of bandages and splints, was maintained and gradually increased ; and on the 20th day the volume of the bone was found evidently diminished, and the fractured portions in good adaptation. The hydriodate was augmented in dose from time to time, and the patient's health continuing to improve, the apparatus which had been firmly bound until then, was removed on the 65th day. The size of the bone being much diminished, and certain eminencies and rugosities, before very perceptible, nearly effaced, on the 85th day the patient began to use his arm. The muscles had contracted adhesions with subjacent parts, and the movements of the limb were long very limited. These became gradually more extensive, and the absorption of the enlargement continued when he was dismissed, so that the difference between the two arms was comparatively trifling.

M. Godard observes, in reference to the medicine employed,—

"Having obtained very remarkable results from the employment of the hydriodate of potash in the treatment of many exostoses situated in different parts of the body, some syphilitic, but the greater part not arising from syphilis, I determined upon trying it associated with bitters in the present case. I have employed it in a great number of old and obstinate glandular enlargements, with some success; but its effect is less certain and more slow in these maladies than in the diseases of the osseous tissue."

Our space forbids our noticing two interesting papers, one upon the Dislocations of the Astragalus, and the other upon the Solubility of various Salts. There is also a code of minute Instructions issued by the Military Council of Health for the prompt treatment of every variety of Asphyxia. Artificial respiration is forbidden under any circumstances as both useless and dangerous—the incipient respiratory movements being aided by repeated slightly alternated compression of the dorsal regions and flanks.

The volume terminates with a report of some of the addresses delivered upon the translation of the mortal remains of Broussais to the Val de Grace. Orations of this description, so frequent in France, may seem somewhat too theatrical to our more prosaic and phlegmatic temperaments; but still we must acknowledge there is something touching in the affectionate remembrance in which that country holds her great men; and we fear we may be sometimes too justly reproached with coldness and indifference in this respect.

